# Restrictive Measures for Children During the COVID-19 Pandemic: Are They Scientifically Supported?

**DOI:** 10.3389/fped.2021.573061

**Published:** 2021-04-22

**Authors:** Nicola Principi, Susanna Esposito

**Affiliations:** ^1^Emeritus professor of Paediatrics, Università degli Studi di Milano, Milan, Italy; ^2^Department of Medicine and Surgery, Pediatric Clinic, Pietro Barilla Children's Hospital, University of Parma, Parma, Italy

**Keywords:** COVID-19, SARS-CoV-2, restrictive measures, imunity, pediatric age (children)

## Introduction

To reduce severe acute respiratory syndrome coronavirus 2 (SARS-CoV-2) circulation and contain the coronavirus disease 2019 (COVID-19) pandemic in most countries, national governments have implemented restrictive measures, such as physical distancing, lockdown, restrictions of movement and border closures, and surveillance strategies. The whole population, including children of any age, was involved in the restrictions. However, it cannot be excluded that many of the measures targeting children, including school closure, may have been unnecessary or even negative based on insufficient scientific evidence (1).

## Role of Children in COVID-19 Pandemic

It can be assumed that the main reason for keeping children at home may derive from the persuasion that children could be an important potential cause of virus diffusion and COVID-19 development in the community, as in recent years repeatedly reported for another viral infection, influenza (2). Moreover, the real role of children and adolescents in this regard is unknown. Epidemiological studies indicate that both the global incidence rate of COVID-19 in children and the prevalence in children with severe disease are extremely low. In the USA, on September 17, 2020, it has been calculated that only 10.3% of all COVID-19 cases were diagnosed in children and that, among them, only 0.2–8% were hospitalized and 0–0.15% died (3). Table 1 summarizes the characteristics of SARS-CoV-2 positive children admitted to the pediatric intensive care units in different countries. Although no data on pediatric intensive care unit admission are available from Africa and Australia (4), these findings suggest that children have only a small role in COVID-19 epidemiology, and that restrictions to their lifestyle habits, including school attendance, are not justified. However, this conclusion might be debated, as the true reasons for the low involvement of children in the present COVID-19 pandemic are not definitively established.

**Table 1:** Characteristics of severe acute respiratory syndrome coronavirus 2 (SARS-CoV-2) positive children admitted to the pediatric intensive care units in different countries.

**Characteristic**	**North America** **(*n* = 48)**	**Europe** **(*n* = 48)**	**France** **(*n* = 27)**	**Italy** **(*n* = 23)**	**China** **(*n* = 8)**
Median age, years	13	6	6	7	9
Underlying chronic disease, no. (%)	83%	52%	70%	50%	25%
Lethality rate, %	4%	6.2%	18%	4%	0

## Discussion

Several hypotheses have been made, but none of them is truly convincing (5). A role has been ascribed to angiotensin-converting enzyme 2 (ACE-2) receptors, the cellular entry receptor for the virus. However, different suppositions have been made, and no definitive conclusion has been drawn. As the density of ACE-2 receptors in the cells of the different body tissues seems to be significantly lower in children than in adults, it was thought that this could condition a lower cytolytic effect of SARS-CoV-2 (6). In contrast, as ACE-2 can also be found in soluble form and children have higher concentrations of the circulating enzyme, it was supposed that soluble ACE-2 could exert a protective activity by blocking the virus before attachment to the cells. Unfortunately, whether and how ACE-2 can induce protection in children is not definitively defined.

Other hypotheses have regarded a more effective innate immunity or the presence of a pre-existing immunity, trained by previous frequent respiratory infections and vaccine administration (7). Generally, compared with adults, healthy children have a stronger innate immune response, with a higher proportion of total lymphocytes, absolute numbers of T, B, and natural killer (NK) cells, and lower proinflammatory cytokines. In adults with severe COVID-19, lymphopenia is common, and this is supposed to be evidence of a poor innate immune response in these individuals. Children could have better innate protection, but evidence that, in many COVID-19 children, the number of lymphocytes is reduced (3) seems to deny this possibility. On the other hand, it cannot be excluded that the small role of children in COVID-19 epidemiology may simply be the consequence of the low exposure of children to the virus (8). After the declaration of the pandemic, children remained generally at home without substantial contacts with infected individuals unless one of the household members had been infected. Theoretically, if children had been exposed to infected persons as frequently as adults, the COVID-19 incidence rates of these subjects could have been different and become nearer to those found in healthy adults.

Regarding the role of children as spreaders, it seems more supposed than proven. To transmit infection, children must shed SARS-CoV-2 in adequate amounts. Generally, viral load is measured by molecular methods, but these do not measure the true amount of the live virus (9). Moreover, the infectious dose is not precisely defined. Finally, it is not established whether viral shedding differs according to age and in asymptomatic compared with symptomatic children. Results of studies in this regard are conflicting. Semi-quantitative real-time polymerase chain reaction results obtained in children hospitalized in a pediatric dialysis unit suggest that symptomatic children pose a significantly higher risk of spreading the virus in the hospital setting, as the viral load calculated in their respiratory samples was ~200-fold higher than that in asymptomatic children (10). Moreover, it was reported that only 250/33,041 (0.65%) of children without symptoms of COVID-19 had SARS-CoV-2 in their respiratory secretions (11). As in adults, it has been found that the virus content in respiratory secretions is greater in subjects with severe disease than in those with mild to moderate symptoms. These findings suggest that the risk of COVID-19 spread by children seems to be very low. Results from other studies could, however, lead to opposite conclusions. It was shown that the duration of viral shedding by asymptomatic children is only marginally lower than that of children with upper or lower respiratory tract disease (12), and that viral load in younger children can be even greater than that evidenced in older pediatric patients (13).

The relevance of SARS-CoV-2 fecal shedding is also completely unknown. In a study in which 10 children with COVID-19 were enrolled, it was shown that eight of them had shed SARS-CoV-2 in remarkable amounts for several weeks, remaining detectable well after nasopharyngeal swabs turned negative. This was considered evidence that the fecal–oral transmission of the infection might be possible (14). However, as in the study, the replicative capacity of the virus was not tested, and whether children may transmit the infection through feces could not be definitively established. However, if viral studies do not solve the problem of the importance of children in the spread of SARS-CoV-2, some epidemiological evidence seems to indicate that children have a minor role compared with adults in this regard. Data collected in different settings suggest that in most of the cases, children are infected by adults, and that the risk of SARS-CoV-2 transmission between children and from children to adults is significantly lower (15, 16).

The introduction of restrictive measures for COVID-19 has deeply modified children's lifestyle behaviors with a reduction in education, socialization, and physical activity (17). Several medical, familial, social, and economic problems have emerged, only partly reduced by specific interventions. It is not precisely defined whether school closure is really effective, at least to reduce severe COVID-19 cases in school children and teachers. A study carried out in Sweden did not find difference in child mortality rate in the 4 months before and after the pandemic declaration with very poor involvement of teachers in severe COVID-19 (18). It is possible that restrictions have inadvertently done more harm than good. Further studies specifically planned to evaluate the role of children in the COVID-19 pandemic are essential. More in depth and precise knowledge on how many children infected by SARS-CoV-2 remain asymptomatic, whether and for how long they can be infectious, and whether there are differences according to age can allow a better approach for children during the COVID-19 pandemic. Further advantages could be derived from studies capable of establishing the immune response of children of different ages to SARS-CoV-2 infection. The evidence that healthy subjects can have stereotypic antibody clonotypes capable of providing protective immunity against viral infections by neutralization deserves attention (19). The identification of those that are naturally protected or who have already developed a protective immune response could favor the mitigation of restriction at least for a select group of subjects.

## Author Contributions

NP and SE co-wrote the manuscript and provided substantial scientific contributions. Both authors have read and approved the final version of the manuscript.

## Conflict of Interest

The authors declare that the research was conducted in the absence of any commercial or financial relationships that could be construed as a potential conflict of interest.
